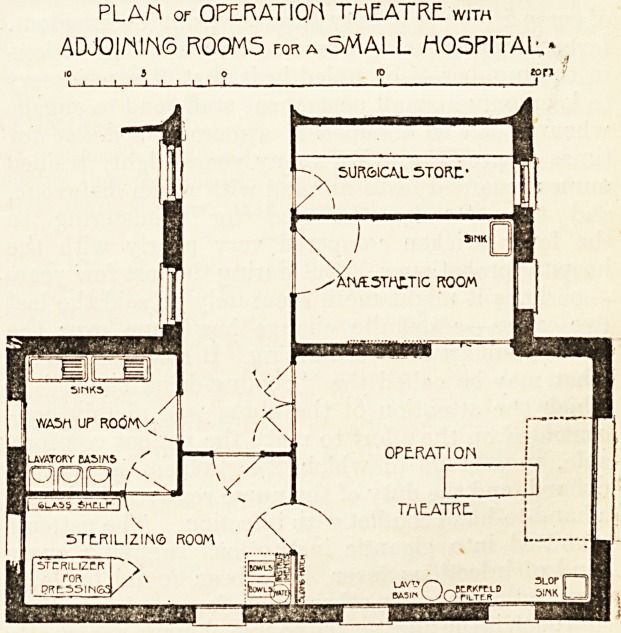# On the Arrangements for the Operation Theatre for a Small Hospital

**Published:** 1908-02-29

**Authors:** 


### ON THE ARRANGEMENTS FOR THE OPERATION THEATRE FOR A SMALL HOSPITAL.

No part of a hospital has undergone such important modifications of recent years as the operation theatre with its appurtenances. In the great hospitals with schools, in place of the big theatre with its tiers of seats for students, we now see four or five small theatres with a limited space set apart for the small number of students who can see something of the work that is going on—and while the size of the theatre itself has much decreased, correspondingly the number of subsidiary rooms has increased.

In small hospitals the provision for operations often consists of only one room, in which, besides the actual operation, the work of sterilising instruments and dressings, administering anæsthetics, and cleaning up after an operation is also performed.

For a cottage hospital, where the work is of a less important nature and also less in amount than in a hospital of, say, 50 to 100 beds, the single room may suffice fairly well—although even in a cottage hospital a separate room for administering anæsthetics is a humane arrangement which should be provided if possible.

The particular class of hospital to which we now wish to direct attention is the provincial hospital of, say, 100 beds, where surgery of an important nature is carried on, and where operations are done in considerable numbers. The arrangements necessary for such a hospital are shown on the plan which has been designed for a small general hospital of 50 beds by Messrs. Young and Hall. The whole department consists of five rooms: (1) Surgical store, (2) anæsthetic room, (3) operation theatre, (4) sterilising room, and (5) wash-up room. All except the first communicate with each other. The anæsthetic room is entered from the corridor by a door of sufficient width to admit an ambulance. From this room the patient passes to the theatre by a sliding door, an arrangement for economising space and facilitating the speedy manipulation of the ambulance. The operation over, the patient is taken away through the doors leading into the corridor.

The theatre itself is 19 feet by 16 feet. In length it might be reduced by a couple of feet, but the actual dimension was controlled by the arrangement of the floor below. The only fixtures in the theatre are a slop sink and a lavatory basin. The necessity of having close at hand means of at once disposing of septic matter is obvious, and a well-devised slop sink with automatic flushing apparatus is the best means of accomplishing this. The basin is a movable one which fits into a metal ring and can be taken away and boiled after being used. Hot and cold sterilised water is laid on to this basin, and can also be drawn by a separate tap into a hand-basin. The sterilising room, which immediately adjoins the theatre, contains a steam steriliser for dressings, two smaller ones for bowls and other vessels, one for instruments and one for water. The four last-named apparatus are grouped together under a hood which communicates with a flue to carry off the steam. A sliding window in the operation theatre is arranged so that instruments can be quickly passed through, and the tap of the sterilised hot-water apparatus is so placed that water can be drawn in the theatre. A glass shelf for dressings completes the equipment of this room. In the wash-up room adjoining are three lavatory basins, each fitted with treadle apparatus for supplying hot, cold, or tepid water through a rose jet fixed over the basin; also two large glazed porcelain sinks with draining slabs all made in one piece.

The walls of all these rooms will be lined with white opalite tiles with hollow internal and rounded external angles. The floors will probably be laid with Terrano, a plastic material made principally with wood meal (a very finely powdered sawdust) and chloride of magnesium. It forms a hard but elastic impervious surface, and is less expensive than Terrazzo; it has the further advantage of not being liable to crack. The doors will all be made of teak, with flush surfaces both sides, and the windows will be of wrought steel enamelled.

The question of the best wall covering for an operation theatre is frequently discussed, and it is often urged that a hard cement finished with paint and varnish is as good, and, of course, less costly than glass tiles or marble. It cannot be too strongly urged that no known form of cement is impervious. Varnished surfaces will remain impervious, but for how long is not precisely known. For economical reasons, however, there is much to be said in favour of varnish and paint. Putting marble on one side as a costly material, and not by any means an impervious one, we have in glass tiles (opalite or the like) a material which, so far as the tiles themselves are concerned, is absolutely im-

PLAN OF OPERATION THEATRE WITH ADJOINING ROOMS FOR A SMALL HOSPITAL.

pervious. It will of course be said that the joints are not, and cannot be made impervious. No doubt this is the case, but when the tiles are well laid the joints are very fine indeed, and the whole surface can be washed down with a hose, like paint and varnish. The latter after repeated hosing may need renewal, but the former surface is not affected except so far as some of the plaster in the joints may be injured.

The warming and ventilation of the theatre will be accomplished by means of a hot-water coil under the sill of the large window, at the back of which fresh air will be delivered by a fan fixed on the outside and worked by an electric motor. Between the fan and the coil will be a screen fitted with a filtering material, on which the greater part of the floating matter in the air will be caught. At the ceiling level will be an exhaust shaft, also fitted with an electric fan, for the extraction of vitiated air. The other rooms will be warmed and ventilated in the same way, except that fans will not be used for driving the air in, force being applied for extraction only.

## Figures and Tables

**Figure f1:**